# Suppression of the High-Frequency Errors in Surface Topography Measurements Based on Comparison of Various Spline Filtering Methods

**DOI:** 10.3390/ma14175096

**Published:** 2021-09-06

**Authors:** Przemysław Podulka

**Affiliations:** Faculty of Mechanical Engineering and Aeronautics, Rzeszow University of Technology, Powstancow Warszawy 8 Street, 35-959 Rzeszów, Poland; p.podulka@prz.edu.pl; Tel.: +48-17-743-2537

**Keywords:** surface topography, surface metrology, measurement errors, spline, spline filter, modelled data, raw measured data

## Abstract

The metrology of so-called “engineering surfaces” is burdened with a substantial risk of both measurement and data analysis errors. One of the most encouraging issues is the definition of frequency-defined measurement errors. This paper proposes a new method for the suppression and reduction of high-frequency measurement errors from the surface topography data. This technique is based on comparisons of alternative types of noise detection procedures with the examination of profile (2D) or surface (3D) details for both measured and modelled surface topography data. In this paper, the results of applying various spline filters used for suppressions of measurement noise were compared with regard to several kinds of surface textures. For the purpose of the article, the influence of proposed approaches on the values of surface topography parameters (from ISO 25178 for areal and ISO 4287 for profile standards) was also performed. The effect of the distribution of some features of surface texture on the results of suppressions of high-frequency measurement noise was also closely studied. Therefore, the surface topography analysis with Power Spectral Density, Autocorrelation Function, and novel approaches based on the spline modifications or studies of the shape of an Autocorrelation Function was presented.

## 1. Introduction

### 1.1. Introduction to the High-Frequency Errors in Surface Topography Analysis

Surface roughness is, from a fundamental prospective, closely associated with many problems strongly related to its tribological performance [1]. Considerations of the measurement process and data analysis of surface topography, as a fingerprint of the manufacturing process, are completely justified that many valuable and accurate information considering material contact, e.g., wear resistance, lubricant retention, sealing, friction, fatigue, can be received directly from its studies. Moreover, the measurement process and data processing have a considerable influence on the results obtained. Highly specialized and precise measuring equipment may not provide a comprehensive evaluation of the surface topography properties when errors in the processing of the received raw measured data occur.

It was assumed that many different factors could create noise affecting the uncertainty in the measurement of surface topography. These include a classification of errors: Those caused by the environment [2] (such as mechanical, acoustics vibrations, electromagnetic interference, etc.), errors caused by the instrument such as internal noise and quantization errors or, consequently, errors caused by the data analysis, e.g., numerical uncertainties and model approximations. One of the typical examples of measurement noise is its representative in the high-frequency domain. The high-frequency noise can be caused by instability of the mechanics with any influences from the environment or by internal electrical noise. Nevertheless, the high-frequency noise, in most cases, is the result of vibration.

The data detection and then minimization of the influence of the measurement errors that occurred during the surface texture measurement process seems to be a compelling issue, especially when accurate assessments of surface topography parameters are reasonably required. The errors that occur while measurement proceeds are often defined as a noise [3], e.g., instrument or instrument white [4], random or random height [5], phase [6], signal-to-ratio [7], RMS height [8] or, simply, measurement noise [9]. Furthermore, the process of removal or reduction of those types of measurement uncertainty is defined as a denoising procedure [10].

The noise could be generated by aperiodic stochastic vibration caused by random factors during the cutting process and distributed throughout the entire frequency domain. Nevertheless, this type of frequency-defined noise cannot be classified as irrelevant and reduced, which is a part of surface characteristics and must be analyzed.

To reduce the noise in the interferometric signal, a bandpass filter with a bandwidth in the range of the object’s velocity was proposed [11]. This is used for the machine learning method based on the least squares support vector model. The influence of noise can be also studied on the fractal dimension of the measured surface topography, as well [12].

At present, the use of sensors is more robust to environmental issues, but the electrical noise in the sensor output can cause a High-Frequency Noise [13] (HFN). Reduction of this type of error can provide a better resolution, which also reduces the bandwidth of the sensor. For White Light Interference-based Atomic Force Microscopy (WLIAFM), the HFN method used in the interference signals from the photoelectric devices and the different light is inevitable [14]. These factors lead to the distortion of the interference signals and reduce the sense of the zero-order fringe positioning method [15,16], applied in the WLIAFM approach. According to the non-uniform distribution of the light intensity, the extracted signal is liable to be skew and asymmetric and to contain a lot of HFN.

### 1.2. State-of-the-Art in the High-Frequency Errors Characterisation

For example, in the case of interference microscopes, the interference signal is composed of the background signal, which is characterized by low frequency, noise signal of high frequency, and the useful signal which needs to be processed separately. Received by the measurement process, signal data can also contain other types of measurement errors (noises). Analysis of relevant elements of surface topography can provide a piece of valuable information on how the manufacturing process has proceeded. Precisely conducted processes of surface topography measurement and data analysis can be crucial to both evaluate the mechanical behavior of “engineering” surfaces and be an integral part of process control. Increasing errors, e.g., those received when a form (F-operator) or noise (S-operator) are separated, can cause classification of properly made parts as a lack and its rejection. Therefore, a proper selection of algorithms (filters) for the definition of an S-F surface (surface obtained after application of an F-operator and an S-operator) is required. Separation of irrelevant components (F-components and S-components) from the results of surface topography measurements can be provided with frequency-based algorithms and procedures. By applying a wavelet transform, different frequency components of the interference signal can be decomposed into a non-overlapping band, which occurs to be an advantage in signal noise separation and signal denoising process [17].

The denoising process that was previously proposed involved various wavelet appliances [18], where the rapid decomposition and reconstruction was presented with the Mallat algorithm [19]. Noise can also be removed using the lifting wavelet by setting wavelet difference coefficients (containing the measurement noise with the correct oriented frequency) [20]. Furthermore, reduction of noises can be applied by the phase evolution algorithm, where wavelet transform combined with soft threshold filtering and homogenization, is used to eliminate the distortion of the interference signal [21]. Additionally, while conducting more comparisons, many filters for denoising of the measurement results from surface texture, e.g., 2RC [22], Gaussian, B-spline, and other [23,24] filters or procedures were present in the past studies. Nonetheless, in terms of noise reduction, when the noise spectrum and signal spectrum overlap, the filters not only lose certain frequency information but also cannot achieve a good noise reduction effect in the reserved frequency domain. On the other hand, time-frequency analysis methods are widely used [25] and the rapid inspection of samples for surface texture or shape measurement is often proposed.

Alternatively, to the direct filter applications, the Empirical Mode Decomposition (EMD) was pioneered by Huang et al. [26] for adaptively representing nonstationary signals as sums of zero-mean amplitude modulation frequency modulation components [27]. This technique became a de facto standard for time-frequency analysis of nonlinear and non-stationary signals, its multivariate extensions are only emerging [28]. An EDM method was proposed for a complex-valued data analysis. It was achieved based on the filter bank interpretation of the EMD mapping and by making use of the relationship between the positive and negative frequency component of the Fourier spectrum [29]. One of the types of measurement noise, white in particular, was characterized by the EDM approach [30].

Another problem in surface metrology is the end effect. Generally, it is difficult to determine the signal filtered at the profile (detail) ends, the problem is called the edge (or end) effect. It is often stated that edge-effect in surface topography filtration seems to be a considerable problem when applying digital filtering. Recursive implementation of Gaussian filter occurs to be a reasonable alternative [31] that Gaussian regression filters, which is a combination of a Savitzky-Golay filter with the Gaussian kernel when using the straight line as regression polynomial delivers the identical result at the centre of the topography and minimizes the edge-effects considerably [32]. For an areal and profile application, it was assumed in the ISO standards, as well [33]. In addition, a two-dimensional discrete spline filter [34] can be used to overcome this disadvantage. The edge-effect can also be reduced when high-order spline [35] approaches are used, which is a typical example of an extension of the spline filtering [36]. Fourteen different combinations of boundary conditions of the spline filter [37], wavelets or its combinations [38] were thoroughly analyzed and compared to provide satisfactory end-data analysis. Areal spline filtering was compared with Gaussian approaches or extended Tetrolet transform [39]. Tetrolet transform is one of the multi-scale analysis methods, which is a non-redundant adaptive geometric wavelet transform [40]. Tetrolet does not suffer from pseudo-Gibbs artefacts while preserving the anisotropic edges and features. Nonetheless, the edge-effect derived from the spline filter by the convolution method is not distinguished, which is partly due to the fact that mean lines at profile ends are supposed to be linear or straight as a boundary condition [41].

Different issues related to non-stylus areal measuring instruments are non-measured points [42] or outliers [43], defined in many cases as spikes [44] or noise-like spikes errors [45]. Such a problem can arise when the surface contains steeply sloped sidewalls, i.e., in structured surfaces. Generally, artificial features may arise if filters are used that are not robust against outliers [46]. Minimization of the influence of this type of noise can be applied with various outlier correction procedures [47,48,49].

Further to the previous comments, very popular in measurements and analysis of surface topography are splines. Advantages of this type of filtering method in contrast to the regular Gaussian approaches are indicated beforehand [50]. A two-dimensional isotropic spline filter [51] is proposed, among other things, for separation of the roughness, waviness, and form components of the surface topography. An appropriate problem was formulated to define the mapping of primary surface data into filtered surface data with a transfer function based on the circularly symmetric low pass Butterworth filter of a given order [52]. Furthermore, cubic spline filtering [53], especially B-splines [54], M-estimator, and Gaussian filtering can be used commonly for decomposition of form features from the results of areal surface topography measurement, cubic spline decomposition was comprehensively studied previously [55]. The process of combining data from several information sources (instruments or sensors) into a standard representational format and the surface topography can be reconstructed using least-square B spline approximation techniques [56]. B-spline functions are entirely appropriate in the characterization of defined as freeform or Non-Uniform Rational B-Spline Surfaces (NURBS) [57]. The linear B-spline functions [58] can be combined with other applications, e.g., Haar function when BLac-wavelet is accomplished [59], two operators (smoothing and error) were applied for computing the coarse mesh and to determine the difference between the approximation and the original meshes [60]. B-spline with Haar scaling functions of 0, 1st, or 2nd order can be quite invaluable when round off errors (also called rounding errors) are minimized [61]. An increase in filtration time might be one of the undue complications. Therefore, the fractional spline filter often called the universal spline filter [62], is calculated by a fast Fourier transform. Some of the transform modifications were also applied to perform the phase-shift calibration [63]. A framework for calibration of areal surface topography measuring instruments, including those employing optical techniques, was performed by ISO standards [64] and has been reviewed previously, as well [65].

### 1.3. Selection of Various Types of Surface Topographies for Noise Analysis Improvement

From a functional point of view, the multi-process topographies which bear traces of two or more processes are becoming increasingly important. Plateau honed cylinder liner surfaces, widely analyzed in the current paper, are representative examples of textured surfaces with networks of micro reservoirs, frequently referred to as dimples, oil pockets or, simply, cavities. Previous discoveries prove that this type of surface texture has excellent sliding properties and an outstanding ability to preserve the oil in its rough topography. The oil pockets or, simply, dimples can provide a microreservoir oil protection, when starved lubrication appears in which surfaces containing dimples have a definite advantage over one-process surfaces, it can be done by laser texturing [66]. The description of the tribological performance of these types of textures is vital. Flawed estimations of select properties, such as the form, undulation, or noise of these types of topographies may cause the rejection of working parts or classify them as missing. These types of elements work with turned piston skirts, and it was discovered that the waviness layout is largely disrupted when surfaces contain curvature, imperfections in the manufacturing process [67] cause many such disruptions. Thus, stratified functional surfaces were analyzed in detail previously [68] with comparisons of various optical measuring techniques for turned topography assessments [69].

Surface topographies produced with laser melting methods may be easily classified within the group of complex textures that make the analysis of measurement results (data) more complicated. They feature significant topographic details in multiple scales, with a mixture of high and low aspect-ratio formations, high slopes, undercuts and deep recesses, especially in lower-density builds. These types of surface topographies are challenging to measure, for example, the general shapes of some topographic features are constructed in various ways, depending on the measurement technology. Moreover, the small cavities may become protrusions, while regular hemispheric shapes (e.g., spatter particles) may seem to be irregular. The comparison of the measurement of this type of surface was not efficiently performed previously. The textures analyzed in this paper have been widely used in mechanical, automobile, and chemical departments due to their toughness, excellent wear resistance, performance stability, and other considerable advantages [70]. The ceramic ground surface was analyzed, and the surface topography ceramic femoral head was previously studied [71], the geometric structure of the rough surface is connected to many engineering problems, e.g., sealing, friction [72], or wear [73,74,75]. Despite that, the engineering ceramic surface is generally different from the metal surface after machining. Therefore, measurements of these types of surface topographies might be considered as severely disrupted. The analysis of milled surface textures is crucial, especially at the end of the milling process, it is one of the most important criteria used to determine the machinability of particular workpiece materials. Many papers focus on conventional milling [76] of easily cut workpiece materials or machining at different workpiece inclination angles. However, not many articles focused on the errors in the computation of surface topography parameters with noise occurrence specifications.

Other types of the analyzed surfaces are isotropic one-process or two-process surface textures, they were studied compressively with the contact of flat surfaces. The effect one-process surface texture has on the relation between contact area and separation depends on the contact area. The impact of the valley surface portion is also important, but less so. The detection and the subsequent removal (or minimization) of the noise errors have not been comprehensively studied either.

### 1.4. Motivation

Except for many papers considering the measurement noise [77,78,79], only a few of them treat the high-frequency noise and its influence on the results of surface topography measurement. Therefore, there is still no full response for the characterization of the errors in the high-frequency domain when the surface topography is measured. Therefore, in this paper, two separate digital processes, detection and suppression (reduction), were provided. For the definition (detection) of the high-frequency errors from the received raw measured surface topography data, the following procedures were proposed: Spectral (power spectral density application), or autocorrelation function analysis, spline modifications of profiles and analysis of a shape of the autocorrelation function.

Furthermore, for the reduction (suppression) of the high-frequency noise, a procedure based on the multithreaded analysis of the results obtained, was suggested. For this approach widely used in surface metrology, spline filters were comprehensively studied, compared, and suggested with the consideration of the high-frequency errors.

From the above, the primary objective of this paper is to propose and compare various procedures for the detection (suppression) of high-frequency measurement noise from the surface topography data of the different types of surface textures. Secondly, verification and an improvement of the widely used surface metrology, various spline filters with minimizing of its distortion influence on the values of surface topography parameters.

To confirm the results obtained, the studies were carried out on the simulated (modelled) data with a procedure for minimization of influence of noise-reduction algorithms on surface topography parameters calculation. To propose conclusions, surfaces measured with different methods (stylus and optical) were considered. However, the results received with both methods were not compared, in which the recommendation of the contact and non-contact measurement processes were not one of the purposes of the paper.

To provide a more comprehensive solution, the analysis was performed with areal (3D) or profile (2D) studies, nevertheless, areal measurement was supplied and profiles were extracted in adequate sections, where suggested approaches and proposed algorithms could be improved. Therefore, in some instances, the profile measurement was indicated rather than a profile extraction process. Moreover, the influence of the direction of the profile extraction was taken into account, especially when commonly-used functions (spectral or autocorrelation) were applied.

In the end, a suggestion of application of spline filters, with caution to the minimization of distortion of the surface topography parameter values, were provided. All of the considered filtration techniques (spline filters) can be replaced by different methods (e.g., plenty of Gaussian, morphological, wavelets) with an application of proposed schemes, suggested for the detection and suppression of the high-frequency measurement errors from the results of surface topography measurements.

## 2. Materials, Measurement Process, and Applied Methods

### 2.1. Analyzed Surfaces

In this paper, the following types of surface textures were analyzed: Plateau-honed cylinder liner topographies; honed cylinder liners with burnished dimples of various sizes—diameters ranging from 0.07 to 0.8 mm and depths between 7 and 100 µm; turned piton skirts; ground; milled; isotropic textures; 60- or 120-angle laser liner texturing surfaces; composite and ceramic surfaces.

Many surface features were analyzed and presented in enlarged (extracted) parts, regardless of the size of the measured detail. Moreover, in many noise-detection procedures, the profile (2D) assessments were defined as potentially more decisive than the areal (3D) analysis [80]. Profile and areal surface topography analysis with specifications of values of surface texture parameters can be increasingly valuable in many engineering appliances [81]. Nonetheless, the proposed noise reduction method (HFN, in this instance) included areal appliances of spline algorithms. Therefore, many results were presented in the profile and the surface areas were comprehensively studied.

Some results of surface topography areal measurements were examined with Free-Of-Dimple (FOD) specifications [80] that the occurrence of some surface texture features (e.g., oil-reservoirs, dimples, valleys, treatment-traces in general) has a significant influence on the process of detection of high-frequency components from the surface topography data. “Free-of-dimple” analysis indicates the areas of surface where deep/wide features, did not occur. This type of analyzed detail is flat in general, waviness and form were also eliminated as a preparation (pre-processing) of the analyzed data.

### 2.2. Measurement Process

The details studied were measured by various techniques, stylus or optical, for providing general proposals for suppression of the frequency-defined errors when the measurement process is carried out differently (by stylus tip or white light interference). Nevertheless, the comparison of the influence of a different measuring method on the high-frequency noise occurrence was not studied in this paper, which was not the main objective of the proposal.

The stylus instrument was Talyscan 150 with a nominal tip radius of about 2 μm. The height resolution was 10 nm, and the measured area was 5 by 5 mm (1000 × 1000 measured points), the sampling interval was 5 µm. The measurement speed was 0.5 mm/s or 1 mm/s and its influence was not the preliminary of this research, in which it was comprehensively studied previously.

**Figure 1:**
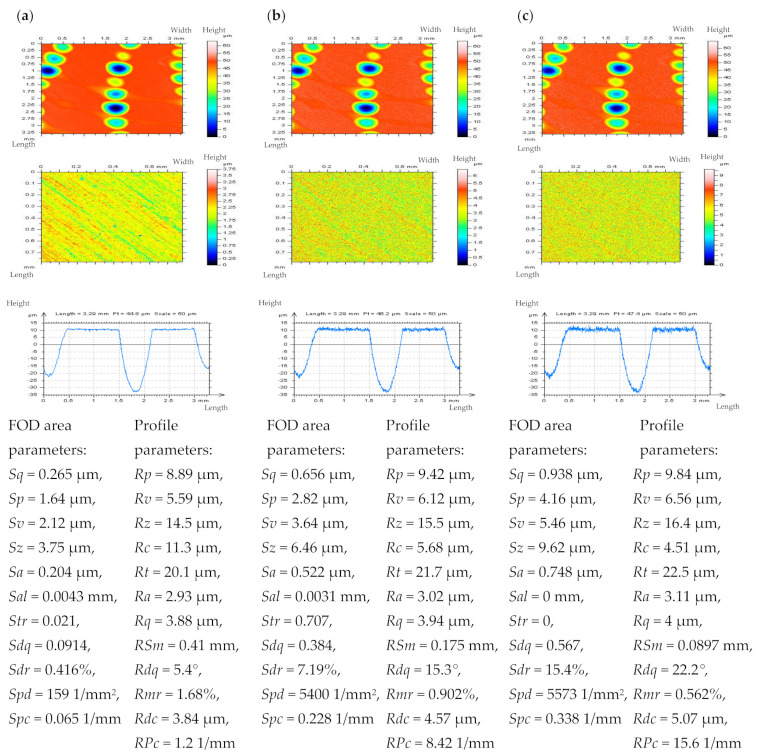
Contour map plots, FOD details, profiles, and select parameters (calculated for FOD areas and profiles) correspondingly, extracted from plateau–honed cylinder liner surface texture measured with stylus instrument with 0.5 mm/s (**a**) velocity and with added modelled noise data with amplitude (*Sq*) equal to 10% (**b**) and 20% (**c**) of *Sq* of the measured details.

The non-contact measurement was completed with the white light interferometer Talysurf CCI Lite. The height resolution equalled 0.01 nm. The measured area was 3.35 by 3.35 mm with 1024 × 1024 measured points. The spacing was 3.27 µm. The effect of sampling on areal texture parameters was not studied in this paper. In Figure 1, cylinder liner surface topographies and profiles containing deep (~60 µm) and wide (~0.6 mm) dimples (valleys) were presented after stylus measurement and after using a modelled noise data added to the measurement results.

All the studied surfaces were provided with an areal form removal process, presented in a previous paper, some published by the author. Received details were flat in general, which did not contain a form (shape or waviness). Moreover, all the details were carefully analyzed for detecting the spikes errors from the measured data. If surfaces contain spikes, they were removed by the thresholding process, described in detail in [49].

It is worth mentioning that the *Sq* of modelled high-frequency noise was equal to 10%, 20%, or, in some cases, to the 30% of the *Sq* value of measured detail, nonetheless, the dimple occurrence caused the noise *Sq* value to influence the non-dimple (FOD) area more than the raw measured surface detail (containing the oil reservoirs). When contour map plots were considered, the noise could be more easily visible when dimples were excluded from the analyzed data (FOD analysis). The high-frequency errors were also easier to detect when a profile was assessed.

The following areal (3D) ST parameters, from the ISO 25,178 standard, were measured and analyzed: Root-mean-square height *Sq*, skewness *Ssk*, kurtosis *Sku*, maximum peak height *Sp*, maximum valley depth *Sv*, the maximum height of surface *Sz*, arithmetic mean height *Sa*, auto-correlation length *Sal*, texture parameter *Str*, texture direction *Std*, root-mean-square gradient *Sdq*, developed interfacial areal ratio *Sdr*, peak density *Spd*, arithmetic mean peak curvature *Spc*, core roughness depth *Sk*, reduced summit height *Spk*, reduced valley depth *Svk*, surface bearing index *Sbi*, core fluid retention index *Sci,* and valley fluid retention index *Svi*.

The profile (2D) parameters, from the ISO 4287 [82], were also considered, as follows: *Rp* maximum peak height of the roughness profile, *Rv* maximum valley depth of the roughness profile, *Rz* maximum height of the roughness profile, *Rc* mean height of the roughness profile element, *Rt* total height of the roughness profile, *Ra* arithmetic mean deviation of the roughness profile, *Rq* root-mean-square (RSM) deviation of the roughness profile, *RSm* mean width of the roughness profile element, *Rdq* root-mean-square slope of the roughness profile, *Rmr* relative material ratio of the roughness profile, *Rdc* roughness profile section height difference, *RPc* peak count of the raw profile.

The effect of the presence of high-frequency noise on the values of surface topography parameters was not studied in this paper, it was considered previously. Nevertheless, some of the parameters, based on the prior studies, were considered as “noise sensitive”, this classification was particularly valuable for the processes of a modelled data noise characterization.

### 2.3. Compared Spline Filters for Noise Suppressions

In order to characterize various types of surface textures, four different kinds of spline filters and functions were described and compared. While considering the classification of a spline function, the spline filters can be roughly divided into non-periodic and periodic schemes [83]. Open (flat) profile filtering is an example of a non-periodic method, while closed (roundness) is a periodic spline filtering method [46]. In short: The non-periodic spline filters can be useful when filtering non-periodic profiles, such as roughness [84]. A simple closed formula cannot give the weighting function of a spline filter. Thus, filter equations are used rather than weighting functions to describe spline filters [46]. In general, splines are low degree polynomials that may be pieced together to form very smooth functions, which in turn may be locally altered to meet any preference [85]. Furthermore, according to the ISO/TS 16610-22 [86], the spline filter has two kinds of boundary conditions: Nature, which is assigned to the non-periodic condition, and the other, classified as the periodic condition [50].

The filter equation for the non-periodic spline filter is as follows:

(1) $$\left(1+\alpha^{4}Q\right)\omega =z$$

where *ω* is a vector of output data values, and *Q* is the matrix as follows [58]:

(2) $$Q=\left[\begin{array}{ccccccccc} 1 & -2 & 1 & & & & & & \\ -2 & 5 & -4 & 1 & & & & & \\ 1 & -4 & 6 & -4 & 1 & & & & \\ & . & . & . & . & . & & & \\ & & . & . & . & . & . & & \\ & & & . & . & . & . & . & \\ & & & & 1 & -4 & 6 & -4 & 1\\ & & & & & 1 & -4 & 5 & -2\\ & & & & & & 1 & -2 & 1 \end{array}\right]$$

and *α* is defined as

(3) $$\alpha ={\left(2\sin\left(\frac{\pi \Delta x}{\lambda_{c}}\right)\right)}^{-1}.$$

Once the sampling interval ∆*x* is small enough, the weighting function *s*(*x*) can be approximated by the continuous function of [85]:

(4) $$s\left(x\right)=\frac{\pi }{\lambda_{c}}\sin\left(\sqrt{2}\frac{\pi }{\lambda_{c}}\left|x\right|+\frac{\pi }{4}\right)\exp\left(\sqrt{2}\frac{\pi }{\lambda_{c}}\left|x\right|\right).$$

The areal smoothing spline filter is widely presented in [34].

In the periodic spline filtering method (e.g., Close Profile Spline Filter—CPSF), the *Q* matrix is defined as follows:

(5) $$Q=\left[\begin{array}{ccccccccc} 6 & -4 & 1 & & & & & & \\ -4 & 6 & -4 & 1 & & & & & \\ 1 & -4 & 6 & -4 & 1 & & & & \\ & . & . & . & . & . & & & \\ & & . & . & . & . & . & & \\ & & & . & . & . & . & . & \\ & & & & 1 & -4 & 6 & -4 & 1\\ & & & & & 1 & -4 & 6 & -4\\ & & & & & & 1 & -4 & 6 \end{array}\right]$$

It is valuable to compare the Open Profile Spline Filter (OPSF) weighting function with those defined for the Gaussian filter, e.g., the Open Profile Gaussian Filter (OPGF). The linear spline filter has two main advantages: There is a largely reduced boundary effect, and it is fitted with an excellent form-following property [87]. The periodic spline filter can be applied to textures in which the form/waviness was not removed, but the HFN decomposition is required, despite the fact that the form-removal process was performed previously. The discrete spline filter was developed in order to avoid the disadvantage of the 2RC filter, i.e., the bias effect falsifying the data under certain circumstances, the presented filter is periodic, has no bias effect, and the filter algorithm is fast and reliable [88].

Cubic spline functions are defined as practical tools for levelling out noise in data [89] and, accordingly, for providing simplicity and efficiency in controlling the process of smoothing [90]. A cubic spline function is a third-degree polynomial in the intervals determined by the consecutive nodes, the nodes of the spline function are defined as measurement points of a profile. Given the example of “noisy data” $f_{i}=f\left(x_{i}\right)$, $i\in \left[0,\;N\right]$, and $x_{0}<x_{1}<\ldots <x_{N}$, the objective is to find a cubic spline smoothing curve $g\left(x\right)$, which would be best for the data analyzed. Moreover, the resulting curve should be adequately smoothed. When $g_{i}=g\left(x_{i}\right)$, the following criterion should be minimized [91,92]:

(6) $$\overset{N}{\underset{i=0}{\sum }}p_{i}{\left(g_{i}-f_{i}\right)}^{2}+\int_{X_{0}}^{X_{N}}{\left[g^{\prime \prime }\left(x\right)\right]}^{2}dx$$

where $p_{i}$ allows controlling the distance for each point individual. When the minimisation equation including the continuity constraints presented above is met, with $g^{\prime }\left(x\right)$ and $g^{\prime \prime }\left(x\right)$ at each $x_{i}$, then

(7) Am=Cg

and

(8) $$g=f-P^{-1}C^{T}m$$

where $f=\left[f_{0},\;f_{1},\ldots ,\;f_{N}\right]$ is a “noisy-data” vector, and $g={\left[g_{0},g_{1},\ldots ,g_{N}\right]}^{T}$ a smoothed vector; $m={\left[m_{0},m_{1},\ldots ,m_{N-1}\right]}^{T}$ with $m_{i}=g^{\prime \prime }\left(x_{i}\right)$; $P=diag\left[p_{0},p_{1},\ldots ,p_{i}\right]$; A is a $\left(N-1\right)\times \left(N-1\right)$ symmetric three-diagonal matrix with $h_{i}/6$, $\left(h_{i}+h_{i+1}\right)/3$, $h_{i+1}/6$ as the non-null elements of the ith row or column. C is a $\left(N-1\right)\times \left(N+1\right)$ three-diagonal matrix with $1/h_{i}$, $-1/h_{i}-1/h_{i+1}$, $1/h_{i+1}$ as the non-null elements of the ith row [89]. The main difference between the regular spline filters and the Cubic Smoothing Spline Filter (CSSF) is that the distance between spline nodes is much larger than the profile sampling interval ∆*x*. Consequently, the conditioning factor of the applied equations system which determines the filter parameters does not depend on the sampling interval. Furthermore, the number of those equations is relatively small [53].

When applying the B-spline filters, the use of an appropriate prefilter prior to sampling may result in a reduced mean square error after reconstruction [93]. A weighting function was derived for zero-order, linear, or cubic spline interpolation. There are certain numerical computations in which the reference surfaces are constructed specifically for waviness or noise extraction. In such cases, the ideal interpolation formula is not practical due to the slow rate of decay of the interpolation kernel. A practical alternative is the reconstruction method using polynomial spline interpolation. The equation is conveniently described as follows [94]:

(9) $$g^{n}\left(t\right)=\overset{+\infty }{\underset{k=\infty }{\sum }}c\left(k\right)\beta^{n}\left(t-k\right)=\beta^{n}c_{\delta }\left(t\right),\;\;\;\;\;\;\;\;\;\;t\in R,$$

where $c\left(k\right)$ are the B-spline coefficients and $\beta^{n}\left(t\right)$ is the central B-spline of n defined as

(10) $$B^{n}\left(t\right):=\overset{n+1}{\underset{j=0}{\sum }}\frac{{\left(-1\right)}^{n}}{n!}\left(\frac{n+1}{j}\right){\left(t+\frac{n+1}{2}-j\right)}_{+}^{n},\;\;\;\;\;\;\;\;\;\;t\in R,$$

where ${\left(x\right)}_{+}=max\left\{0,\;x\right\}$. The B-spline function $B^{n}\left(t\right)$ can also be constructed by a repeated convolution of a B-spline of order 0:

(11) $$B^{n}\left(t\right):=B^{0}\times B^{0}\times \ldots \times B^{0}\left(t\right),$$

where $B^{0}\left(x\right)$ is the indicator function in the interval $\left[-\frac{1}{2},\frac{1}{2}\right)$.

The symmetrical property of the B-splines incurs phase shifts of the spline filter. That is an essential characteristic of the phase-correct profile filters. It is necessary to reconstruct the fractional splines and to symmetrize their support [70].

The B-spline wavelets, e.g., Polynomial Spline Wavelet Filter (PSWF) [95] have the following properties: Bi-orthogonality, compact support, smoothness, symmetry, proper localization, a simple analytical form in the spatial/frequency domains, and efficient implementation. Moreover, B-spline scaling functions are the preferred choice in graphic applications. The B-spline wavelet can be defined as follows:

(12) $$\psi_{m}\left(t\right):=\underset{k}{\sum }q_{m,k}N_{m}\left(2x-k\right),$$

where

(13) $$q_{mk}=\left\{\begin{array}{c} \frac{{\left(-1\right)}^{k}}{2^{m-1}}\sum_{l=0}^{m}\left(\begin{array}{c} m\\ l \end{array}\right)N_{2m}\left(k+1-l\right),\;\;\;\;\;\;\;\;\;\;\;0\leq k\leq 3m-2\\ 0 \end{array}\right.$$

and $N_{m}$ is the mth order B-spline. Since this is only a linear combination of B-splines, the *B*-wavelet also has a compact support. From this mother wavelet $\psi_{m}$, the following wavelet function that exhibits the translation and dilation of $\psi_{m}$ may be constructed [96]:

(14) $$\psi_{ij}\left(x\right)=2^{\frac{i}{2}}\psi_{m}\left(2^{i}x-j\right).$$

In B-spline wavelet filtering, the exact curve representation can be computed in a stable and highly efficient manner. The approximation method begins with the correct solution and the minimization of its complexity by means of degree reduction and knot removal. The proposed degree reduction method [97] is more efficient than other approaches, especially in terms of power and computational efficiency. Moreover, it guarantees approximation within tolerance throughout the entire curve, rather than being confined to sample points.

The bi-cubic splines which include knots within a set of measured data and are formed in the least-square sense, such as the Cubic Spline Outlier Filter (CSOF) [98], may be used as an alternative for outlier detection and filtration processes. Using the B-spline, the bi-cubic spline can be formed as follows:

(15) $$s\left(x,y\right)=\overset{g}{\underset{i=-3}{\sum }}\overset{h}{\underset{j=-3}{\sum }}c_{ij}M_{i}\left(x\right)N_{j}\left(y\right)$$

where $M_{i}\left(x\right)$ and $N_{i}\left(y\right)$ denote the normalised cubic B-spline defined on the knots $\lambda_{i}$, $\lambda_{i+1}$,…,$\;\lambda_{i+4}$, and respectively $\mu_{j}$, $\mu_{j+1}$,…, $\mu_{j+4}$, this complication results in the computation of the coefficients $c_{ij}$ as the solution including the least amount of squares of the $m\times \left(g+4\right)\left(h+4\right)$ system

(16) $$\overset{g}{\underset{i=-3}{\sum }}\overset{h}{\underset{j=-3}{\sum }}c_{ij}M_{i}\left(x\right)N_{j}\left(y\right)=z_{r},\;\;\;\;\;\;\;\;\;\;\;\;\;r=1,2,\ldots ,m$$

which can be written in matrix form as follows:

(17) Ac=z

where the element c is the coefficient $c_{ij}$, z are the values of $z_{r}$, and A is the Kronecker product of two-band matrices of small size $m_{1}\times \left(g+4\right)$, and respectively $m_{2}\times \left(h+4\right)$. Thus, the computation time may be substantially reduced when the number of data points and knots is large [99].

### 2.4. Proposals of Procedures for the Detection of High-Frequency Errors from the Raw Measured Data

#### 2.4.1. Detection of Noise with Power Spectral Density Applications

Noise detection, in this case, HFN, is a demanding and problematic task. One of the proposed solutions is to analyze the Power Spectral Density (PSD) graph. In its two-dimensional form, PSD has been designated as the preferred means of specifying the surface roughness on the draft international drawing standard for surface texture [100]. However, the method of the variance of the PSD estimate reduction should also be proposed [101]. The process of dry machining, including cooling using the Minimum Quantity Cooling Lubrication (MQCL), has proven that it is possible to characterize the turning with regard to the applied cooling methods, using PSD. This is achieved by the qualitative and quantitative comparison of this function for inspected surfaces. Furthermore, the PSD analysis enabled a determination of the amplitude of the feed component, which was observed for higher ranges of feed. For low feed values, cutting tool vibrations and tool edge wear were dominant on surfaces and, respectively, the results based on the PSD analysis can be useful for the identification of surface damages and surface quality [102]. Moreover, the amplitudes of the analyzed surfaces may be approximately defined for different feed values. The process proved that the accurate determination of surface morphology using Scanning Force Microscopy (SFM) imaging and PSD analysis of surface roughness is exceptionally challenging. Furthermore, it is easily affected by experimental parameters, such as the scan speed and feedback parameters [103]. PSD was performed on topography data from a range of optical surfaces of varying quality and manufacturing techniques allowing direct comparison of metrology data obtained by instruments with different spatial bandwidths [104]. In some cases, the PSD enabled the derivation of the surface roughness and thus provide useful information on characteristic features which compose the microstructure of the films and, particularly, as for optical thin films PSD can support optimization of the obtaining processes with the view of reducing scatter losses in thin-film optical coatings [105,106]. Changes in PSD function during engine operation were also analyzed [107] and found that the shape of the cumulative spectrum graph is strongly influenced by the cylinder deep valleys width [108]. The utility of the PSD is that it contains statistical information that is unbiased by the particular scan size. Moreover, some of the recent analytical models and numerical simulations make predictions for functional properties, such as macroscopic contact properties such as stiffness, contact area, and adhesion, based on the PSD of a surface and therefore, generally, the surface roughness can be thoroughly evaluated by the frequency spectral analysis, some examples were widely presented previously [4].

Extensive research proved that the “visibility” of HFN by means of the PSD analysis depends on the size of the surface topography features [109], e.g., the valley diameter and the depth of the plateau-honed cylinder liners with additionally burnished oil pockets [110]. The density (number) of treatment marks also affects noise recognition and visual analysis.

**Figure 2:**
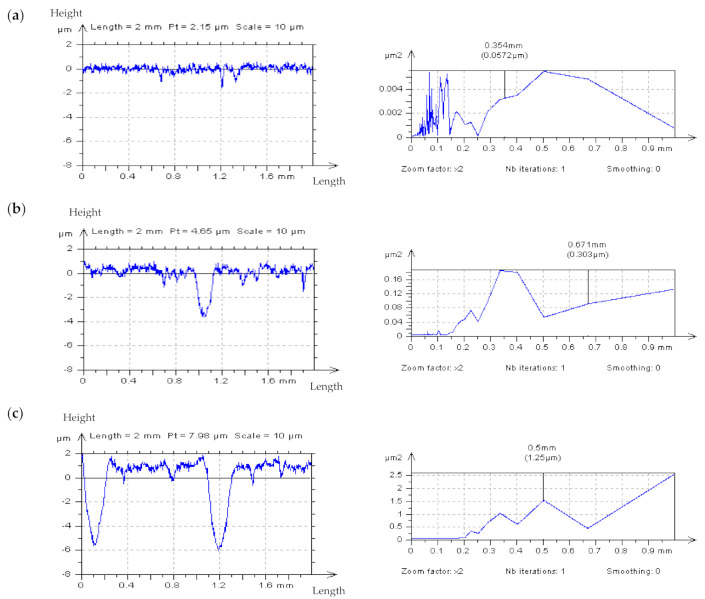
Measured with stylus instrument at 0.5 mm/s speed profiles and their PSDs, respectively, extracted from the plateau-honed cylinder liner texture, examples provided for (**a**) non-valley, (**b**) one-valley, and (**c**) two-valley profiles.

In Figure 2, certain profiles from various areas of the plateau-honed texture were presented. When the profile did not contain dimples (a), the HFN was instantaneously noticed (the small-scale frequencies were discernible). However, if features such as dimples or scratches appeared (b) noise detection became exceedingly difficult. Lastly, when the profile contained more features (usually more than 2 in 2 mm-length profiles) the errors became virtually undetectable (c). The influence of the valley diameter and depth on determining the presence of HFN in the raw measured data has not been studied. The primary purpose of the FOD analysis at this stage of research is to consider the nonfunctional details in order to simplify the analysis of profiles and their PSDs. Nonetheless, the influence of feature size on the process of noise detection should also be addressed in further studies.

#### 2.4.2. Noise Suppressions with Spline Modifications of Surface Texture Profiles

Another method based on the profile detection of HFN is the Profile Spline Method (PSM). In this novel approach, the elements located outside of the valleys of the profile were extracted from the whole profile and merged. In this case, the profile does not have to be a FOD profile, and the number/density of the valley is not necessary. The HFN occurrence was noticeable only when out of-dimple parts of the profile were considered. Therefore, dimples or other features (e.g., valleys or scratches) were omitted.

PSM can be a practical and reasonable alternative for an “only partial” (out-of-dimple or out-of-valley) profile analysis. This approach may be especially useful for topographies containing deep or wide dimples (or other marks), in which the number (density) of this feature is relatively small (usually from 1 to 3 dimples in the profile of a length ranging from 4 to 5 mm).

In some cases, the FOD and PSM methods may be used interchangeably considering that both procedures contain the non-deep-feature elements of the measured detail. PSM is especially valuable for textures in which the non-dimple areas are relatively small (smaller than the diameters of the valley or other features).

Some of the spline methods were applied in order to characterize the two-process profiles. The procedure of distinguishing between plateau and valley components was used. Various methods based on the separation between the plateau and valley regions are presented, which allows an independent functional analysis of the detected features. Previous research has considered the properties of the measured extracted details, not the effect of measurement errors with the assessment of selected (non-feature) parts. Moreover, when the form/waviness components are not removed from the analyzed data, the recognition of thresholds and separation is consequently impossible. The occurrence of the form/waviness affects significantly both methods and, consequently, the value of the thresholding approach is difficult to be defined. Additionally, some of the features (dimples) cannot be entirely excluded when a form was not eliminated previously. It, simultaneously, resulted in an inconclusive detection of the HFN with an application of the PSD analysis.

**Figure 3:**
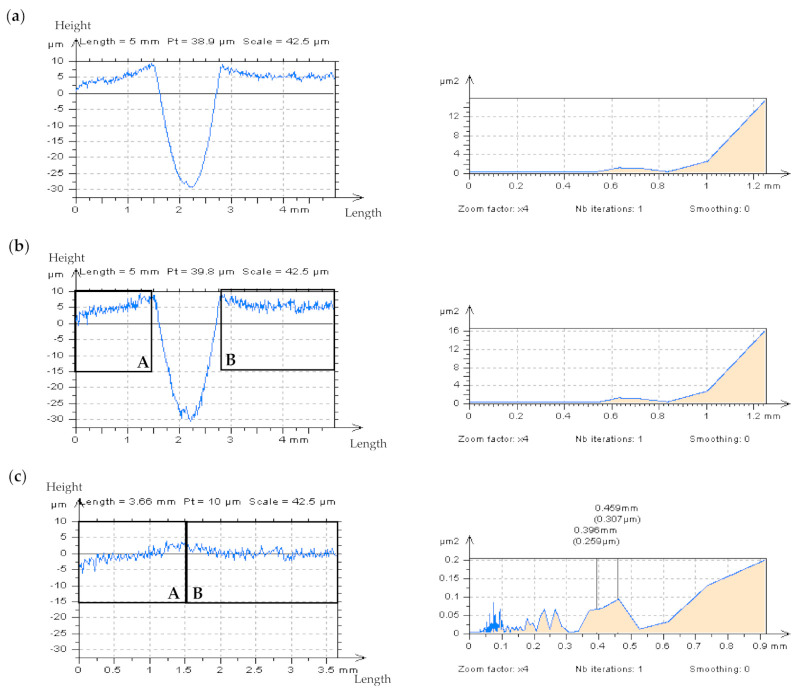
Profiles and their PSDs respectively decomposed from the plateau–honed cylinder liner surface texture containing deep/wide dimple with stylus measuring 0.5 mm/s (**a**) and with simulated high–frequency noise (**b**) with *Sq* equal to 20% of *Sq* of the assessed profile (**b**) and AB–profile (**c**) created using PSM.

In Figure 3, the results of the application of the PSM approach were presented with the characterization of the plateau-honed cylinder liner surface, which contains additionally burnished dimples. The measured profile (a), was divided into three separate parts—two containing out-of-feature characteristics (the A and B details), and the third including a valley element, all presented in (b). In the reconstructed AB profile (c) created with a PSM scheme, the high-frequency noise can be directly observed with the PSD graph. Generally, the PSM approach is more valuable when no differences between the PSDs of measured detail and after adding a modelled noise (between the PSDs from (a) and (b) in Figure 3) are observed and, simultaneously, the dimples (valleys) are omitted.

#### 2.4.3. Definition of Noise by the Analysis of the Shape of Autocorrelation Function

In order to characterize the surface topography, an analysis of the Autocorrelation Function (ACF) was proposed. The ACF assessment provides relevant information about the autocorrelation length and its properties as a function of surface irregularities pitches since it corresponds with the wavelength of the surface topography irregularities. When comparing the PSD and the ACF, the ACF is particularly convenient for the examination of irregular surfaces. At the same time, the PSD is sufficient for the analysis of periodic surfaces, such as turned or milled textures. When considering profiles, the ACF is simply a plot of the correlation coefficient between the surface profile and that same profile shifted in space by a given amount [111].

**Figure 4:**
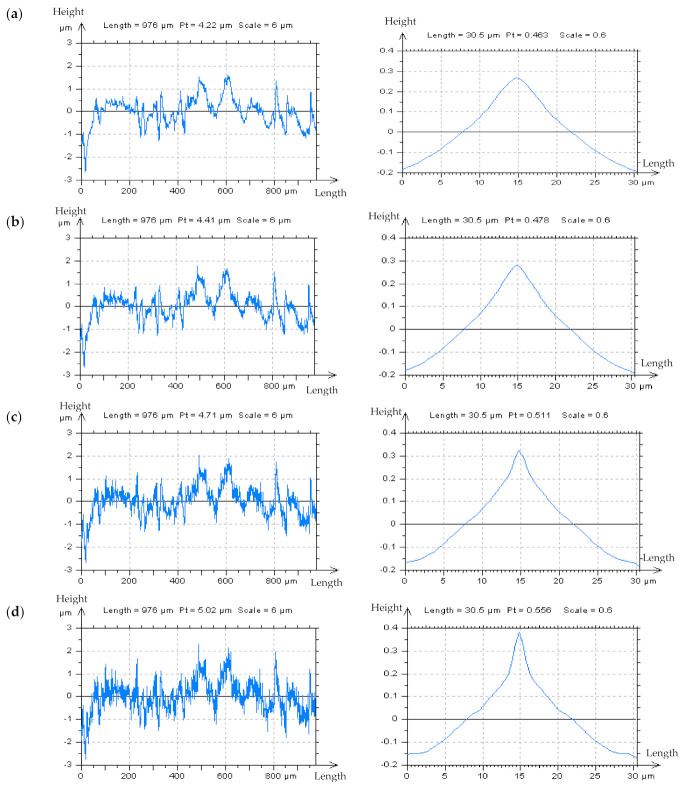
The profiles and their respective centre–parts of their ACFs, measured with stylus instrument of turned details, measured at the speed of (**a**) 0.5 mm/s and with added generated noise with *Sq* equal to 10% (**b**), 20%, (**c**) and 30% (**d**) of *Sq* of the analyzed detail.

In this present work, it was studied if the analysis of the ACF centre shape can provide additional information about the HFN occurrence. When the amplitude of high-frequency noise increased, the ACF centre shape transformed considerably. The angle value of the ACF centre significantly decreased. Figure 4 contains an example of turned detail profiles with an ACFs graph. The initial assumption was that the presence of HFN would cause a considerable disparity in the centre edge of ACF, the place of the highest value of the analyzed function. In most cases, the angle changed from obtuse to concave. Correspondingly, the value of the profile parameter *Pt* increased.

The analysis of the centre shape (except the angle) of ACF was another proposal. Even if a thorough evaluation of this function’s centre angle is performed, it is never independently unambiguous. Therefore, the ACF graph, with its centre-part (defined as approximately 0.050 mm) was studied separately.

**Figure 5:**
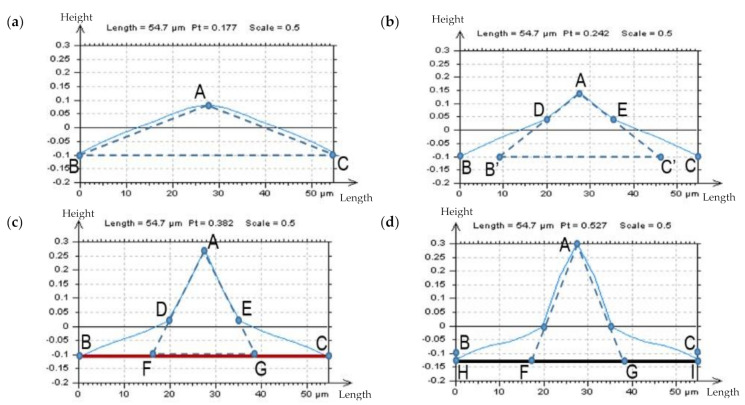
The method for a partial–detection of the HFN from measurement results of turned detail, based on the ACF diagram analysis. ACF diagrams of profiles with no HFN (**a**) and containing small (**b**), medium (**c**) and a lot (**d**) of HFN.

The centre’s angle was defined as the BAC angle (Figure 5). For profiles that do not (probably) contain the HFN, the values of their ACFs centres increased (almost) linearly between the B and A, and between the C and A points (a). When the HFN appeared, the values of ACF increased more rapidly. Furthermore, the D and E points were easily defined. Those points are the tangent positions of the B’A and C’A straight lines, according to the ACF chart (b). Once the HFN recedes (or its presence becomes negligible) the D and E, as well as the B’ and C’ points (points located on the straight line formed by the B and C points), also disappear and are irrelevant in the assessment of the noise occurrence. When the amplitude of modelled high-frequency noise increases the concavity of BAC (and simultaneously of B’AC’), the angle can be observed (c). The process also proved that when

(18) $$\left|BB^{\prime }\right|+\left|CC^{\prime }\right|>\left|B^{\prime }C^{\prime }\right|$$

or

(19) $$\left|BF\right|+\left|CG\right|>\left|FG\right|$$

or, as an alternative

(20) $$\left|HF\right|+\left|GI\right|>\left|FG\right|$$

then the HFN should be significantly reduced (d) from the raw measured data.

**Figure 6:**
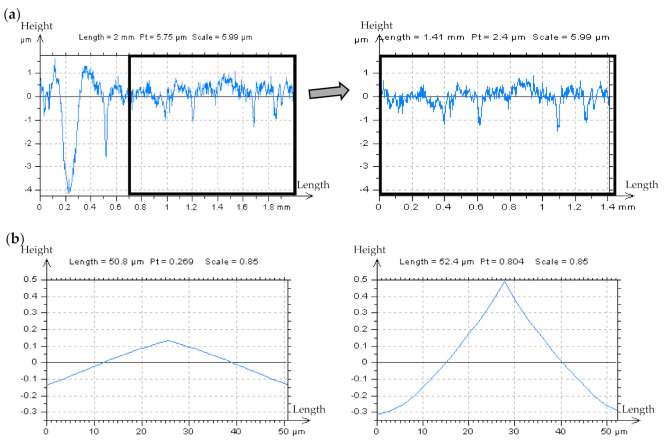
The profiles (**a**) and their respective centre–parts of the ACFs graphs (**b**), extracted from the plateau–honed cylinder liner surface texture with additionally burnished dimples, measured with the stylus technique at a speed of 0.5 mm/s.

When studying the plateau-honed cylinder liner surface, it becomes clear that the analysis of the ACF graph is also required for the non-dimple areas (3D) or profiles (2D), an example of this is visible in Figure 6.

When considering the non-dimple or non-valley profiles, the centres of the ACFs were not acute (the diagram includes an obtuse angle). However, when the profile contains dimples (valleys), the concave angle is plainly visible. This approach is called the Autocorrelation Function Centre-Shape Method (ACF-CSM).

## 3. Results and Discussion

### 3.1. Resolving Problems in the Extraction of Irrelevant Features from Surface Textures

**Figure 7:**
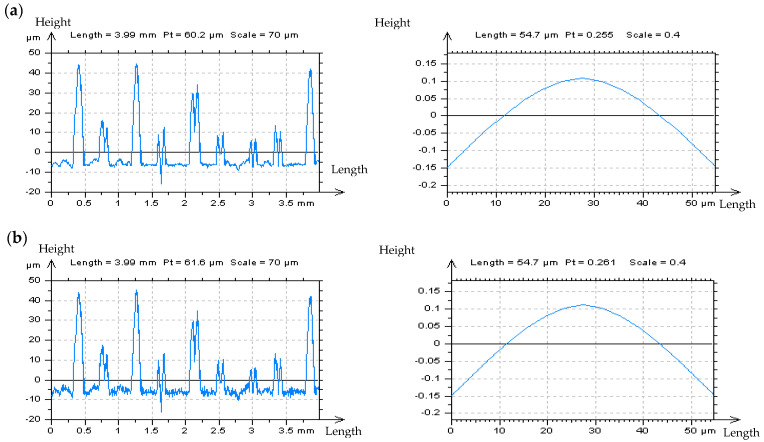
The profiles and their ACF centre–parts, decomposed from laser-textured surfaces, measured with the stylus technique at the speed of (**a**) 0.5 mm/s and after adding a modelled high-frequency noise with *Sq* equal to 20% of the *Sq* of considered detail (**b**).

The problem concerning the detection of the HFN in the results of measured data increases when a flat (FOD, out-of-feature) surface is unachievable. Customarily, when surfaces contain curvatures (e.g., form, waviness), the detection of the HFN is impossible with PSD or ACF applications (description in Figure 7). Laser textured surfaces are examples of these types of topographies. Therefore, the following procedure, simply called the Multithreaded Approach (MA) is advised, which enables detecting of the HFN in the surface topographies of the measured data of the laser-textured details:

(i) Observe the difference in the isometric view of the measured data.
(ii) When the difference in point (i) is not visible, examine the non-plateau and non-valley details.
(iii) When the difference in point (ii) is not visible, extract some non-peak and non-dimple profiles.
(iv) An analysis of the PSD or ACF of the profiles from point (iii) proves to be valuable for HFN detection.
(v) If the above points, (i) to (iv), are ineffective and give no appropriate response, an analysis of an enlarged and extracted centre-part of the ACF of the non-valley/non-peak profile is necessary. When the frame of the received function is accelerating near the maximum value (“1”) of the ACF, the HFN occurrence can be observed.

From the above (MA) approach, some clarifications must be defined. First of all, in some cases, it is possible to observe an HFN with areal or profile data with isometric view studies. Nevertheless, HFN can be difficult to follow when selected profiles are not considered. Therefore, further MA steps must be performed.

**Figure 8:**
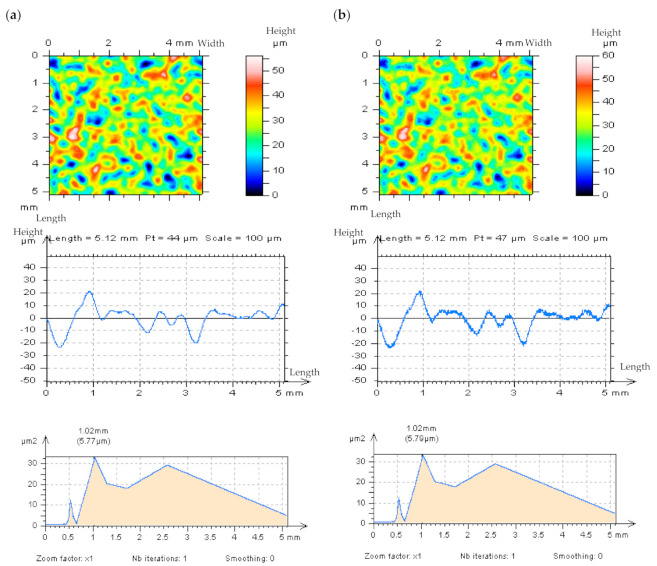

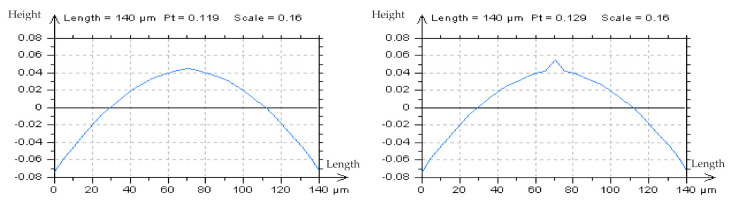
The isometric views of ceramic surface topography, their extracted profiles with PSDs and ACFs, respectively, measured at the speed of 0.5 mm/s (**a**) and after adding a high–frequency noise with *Sq* equal to 20% of the *Sq* of measured detail (**b**).

When considering ceramic surfaces, the detection of the HFN with a PSD graph analysis proves to be entirely ineffective. The noise frequencies are not recognizable in the PSD graph (examples of ceramic textures descriptions are presented in Figure 8. Similar results were obtained for ground details) and the PSD analysis does not enable the detection of the HFNs from the measurements. The noise is easily noticeable during optical profile studies than its PSD graphs. Nonetheless, the assessment of profile PSDs do not illustrate that the HFN exists within the measured data. An analysis of the ACFs for both areal (3D) and profile (2D) measurements also does not prove that the HFN occurred.

**Figure 9:**
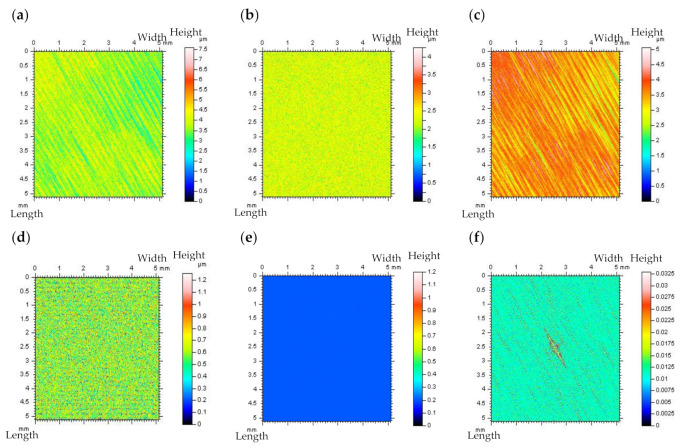
Ground detail measured by a stylus instrument at the speed of 1 mm/s (**a**), and its NS defined by FFTF (cut–off = 0.025 mm) (**b**) with the texture after the HFN denoising (**c**), thresholded (0.13–99.87%) NS (**d**), ACF of NS (**e**) and thresholded ACF of NS received by the FFTF method (**f**); all figures are presented in contour map plots.

The removed data (the results of digital filtration as an S-operator [112]), defined as “Noise Surface” (NS), should only contain the required frequencies, which in this case are high frequencies. Optical analyses of the NS contour map plots do not always allow the detection of all non-noise features. Therefore, the threshold method, precisely described in [49] has been applied, the non-noise (low frequency) features can be more easily identified when using this approach. The threshold method can also be used for areal ACF analysis. Figure 9 illustrates an example of ground topography—the measured surface (a) was filtered by the Fast Fourier Transform Filter (FFTF) approach [113], and surface texture NS (b) was obtained, as an effect of the process of HFN removal (c). The optical analysis of NS did not confirm the presence of non-noise features in NS, created by the application of the FFTF scheme. Moreover, no surface treatment traces were found on the NS contour map plot.

Nonetheless, when the threshold method (from the points of material ratio equal to 0.13% and 99.87%) was applied, some non-HFN elements were established (d). Additionally, the height difference between material ratios from the range of 0.13–99.87% is equal to 6 standard deviations of surface amplitude, for the random surface of the Gaussian ordinate distribution. Previous research also shows [49] that when the height of the areal cylinder surface is limited to material ratios between 0.13% and 99.87%, the areal (3D) surface height is similar to that of the profile (2D). Material ratio curves were presented in many research studies [114]. On the graph of ACF, as defined for NS, the non-noise components (e) were initially observed. Nevertheless, the application of the threshold method proved that some non-noise features were achieved (f). Therefore, the proposed algorithm (FFTF) should not be considered for removing (or substantially reducing) the HFN from the results of the ground textures measurements data. Both the NS and ACF characterization methods, based on the optical analyses and identifications of non-noise features, are particularly relevant in the process of detecting high-frequency errors from the measured data. Nonetheless, the application of FFTF is useful in the detection of the HFN for other types of surface textures, such as ceramic or composite textures which lack scratches (valleys). The application of the FFTF algorithm, with a cut off equal to 0.025 mm is convenient for reducing the HFN from the measured data.

The noise reduction procedure can be performed efficiently when the contour map plot of the surface after the denoising process is directly observed. The application of FFTF for the detection and reduction of HFN caused a relatively small difference (or was otherwise negligible) in the case of the same details measured and with modelled high-frequency noise. Likewise, the smallest differences were found in places with the more radical improvement of the applied algorithm. When the amplitude of the removed data is relatively small (*Sq/Rq* of received NS is smallest than 10% of *Sq/Rq* of the analyzed data) or negligible, then the process of removing the HFN is not required. Defining the “noise surface” [73], it was found that the biggest amplitude of HFN was identified as the largest amplitude of the removed (by S-filtering) data received. For this type of surface, the FFTF noise extraction gave the desired results. It is a valid solution for detecting noise data using the FFTF application.

**Figure 10:**
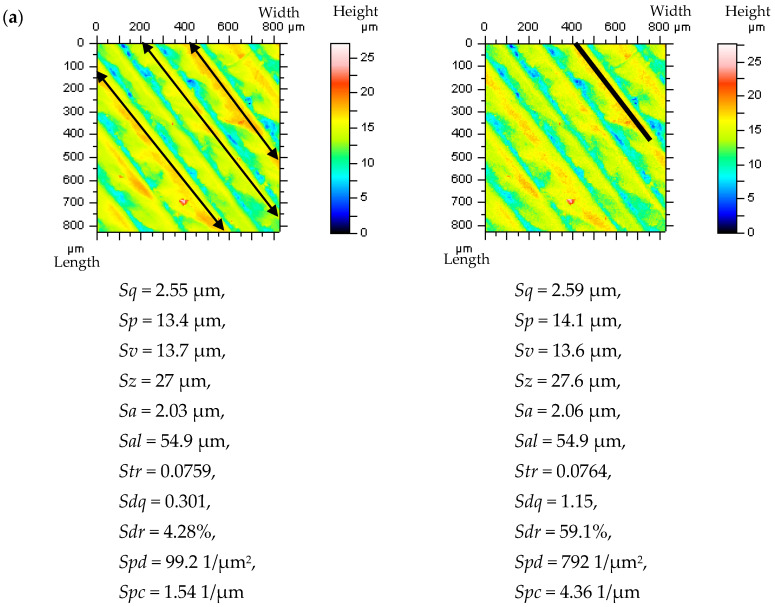

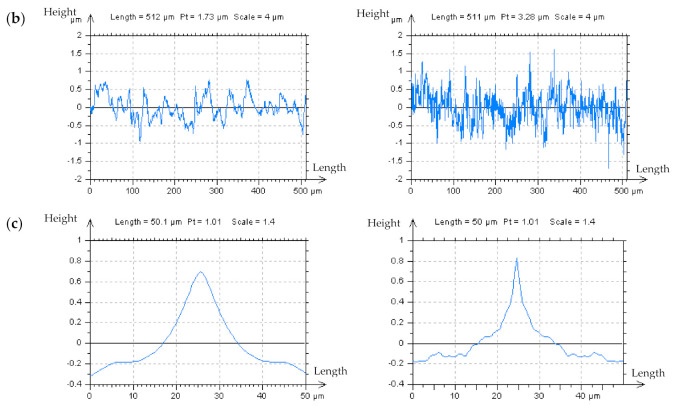
The contour map plots of turned surfaces and their ISO 25178 [86] parameters (**a**), extracted TTPs (**b**) and their ACFs (**c**) of instrument topographies, measured by a stylus method with a speed of 1 mm/s and after adding a modelled noise with *Sq* equal to the 30% to the *Sq* of analyzed surface texture, respectively (from **left** to **right**).

For periodic surfaces, such as the turned, ground, or laser-melted textures, the Treatment-Trace-Profiles (TTP) approach is suggested. When the TTP method is applied, the changes in the ACF shape (increments in the centre part of the function) are more easily detected. Furthermore, the presence of HFN in the measurement results can also be easily detected by analyzing the ACF, as defined for the plateau-part profiles. For all of the measured turned details, the PSD analysis is not a decisive factor in the process of HFN detection. The TTP method is based on the extraction of profiles along the treatment traces. An example of this type of profile extraction is presented in Figure 10. Moreover, the direction and location of the profile layout were presented, being in accordance with the proposed approach (a).

Considering the surface topography parameters, the biggest differences when HFN occurred were observed for the *Spd* parameter. All the height parameters (except for the *Sv*) increased when the noise existed. Both hybrid parameters (*Sdq* and *Sdr*) increased more than 300% and 2000%, respectively. In general, the values of the hybrid and feature parameters mostly increased when the HFN amplitude emerged.

The PSM scheme can also optionally be applied in order to detect high-frequency measurement errors. However, it would require much more digital operation. Additionally, it may cause severe errors in the subtraction of plateau parts of the profile, as opposed to the ACF assessment using the thresholding method. The TTP methods are not particularly useful for the surface topographies not containing the treatment traces, e.g., for ceramic or composite surfaces, which are isotropic in general. Moreover, the detection of high-frequency measurement noise using PSM or TTP might be extremely demanding for isotropic textures. Furthermore, the assessment of NS as defined by FFTF filtering (with the proposed cut-off of 0.025 mm) is rather versatile.

### 3.2. Reduction of the High-Frequency Errors with Comparisons of Various Spline Filters

A thorough evaluation of the procedures (algorithms) applied to reduce measurement errors (especially those of a high-frequency), and their effect on the surface texture data analysis and parameter calculation, is possible with an assessment of the components removed from the measurement results. This removed component is a Noise Surface (NS), defined in the high-frequency domain. All of the proposed methods, including the analysis of NS and its ACFs (with thresholding methods), PSDs, or surface topography parameters, constitute a practical alternative. Consequently, it is possible to compare various methods based on the spline function, e.g., spline filters with various specific purposes, by characterizing the NS.

**Figure 11:**
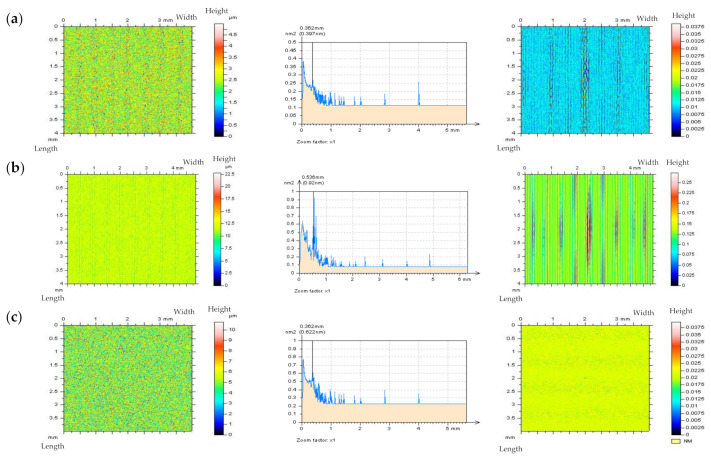

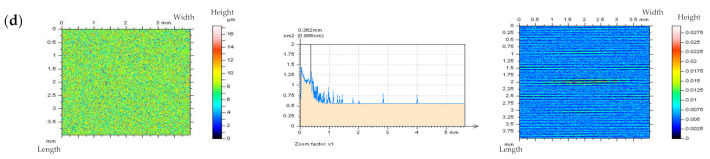
NSs, their PSDs, and threshold ACFs, respectively, described for the laser liner textured surface measured with an optical instrument, defined by: (**a**) CSSF, (**b**) OPGF, (**c**) OPSF, and (**d**) CSOF method; cut–off = 0.025 mm.

It was found that PSD characteristics of NS did not always provide an expected response, especially in cases of extracting solely the noise frequencies (high frequencies). The analysis of the PSD plots (Figure 11) of laser-liner textures proved that the application of the CSSF method caused the deletion of the non-HFN components from the measurements. In the analyzed details, the distance between laser-created features (liners) equalled 0.5 mm. Some non-noise elements are located in NS with a 0.5 mm frequency, which is visible in the PSD graphs (a). Moreover, the noise amplitude is higher in laser formed areas and the threshold ACF graph. Similar effects occur when applying OPGF (b). In both of the filter appliances, the thresholding method for ACF application is a potentially decisive solution.

**Figure 12:**
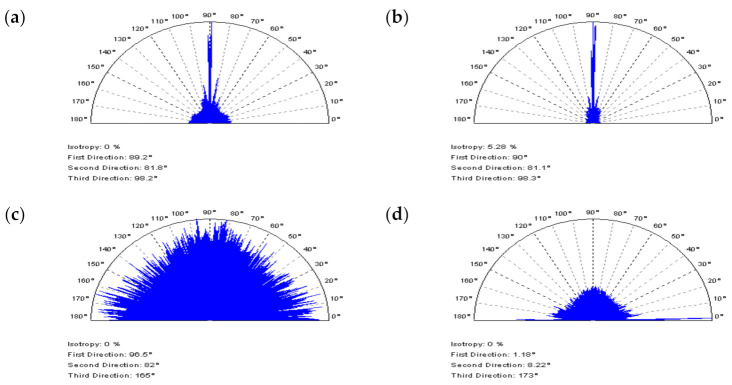
Texture direction graphs of NSs obtained for the laser liner textured surface after the application of (**a**) CSSF, (**b**) OPGF, (**c**) OPSF, and (**d**) CSOF.

Notwithstanding, the PSDs of NS created by applying the OPGF approach illustrate that the features with periodic frequencies (e.g., 0.5 mm) are located on the graph. There are no treatment traces on the contour map plots of NS received by OPSF (c) or CSOF filtration (d). Additionally, some periodic features can be established for their threshold ACFs. Nonetheless, the non-noise components obtained by the CSOF filtration are more noticeable. The NS defined for the removal (reduction) of HFN from the measurement results should be non-periodic, generally isotropic. Therefore, the analysis of the texture direction plot (Figure 12) proves to be of value. In some cases, an analysis of the values of angles, calculated by the software, gave the most intuitive responses. The OPSF has the most direct and desired results of the extraction of HFN from the laser-liner textured measurements, amongst all of the compared spline filters. NS was isotropic and, consequently, its dominant direction on the texture direction graph is unnoticeable, and the periodicity on the ACF is essentially negligible (c).

**Figure 13:**
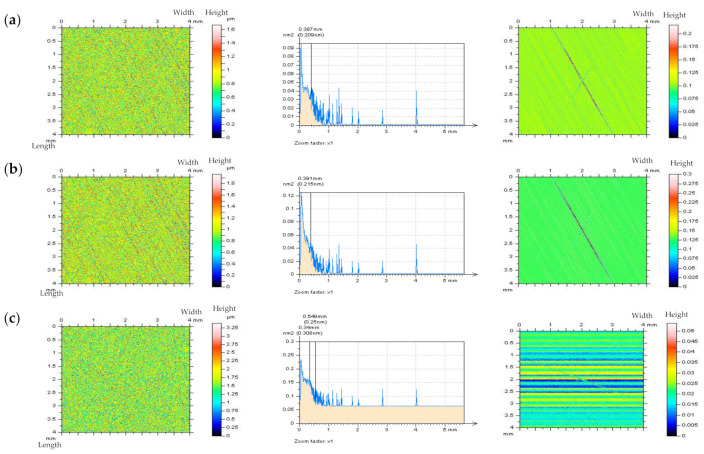

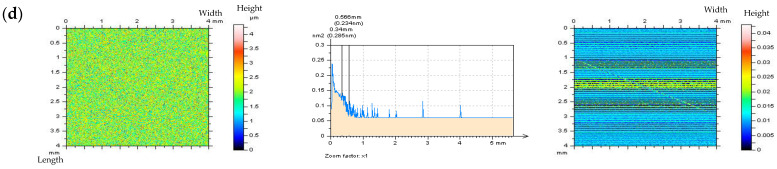
NSs, their PSDs, and threshold ACFs correspondingly, defined for the milled surface texture measured by an optical method, received by: (**a**) CSSF, (**b**) OPGF, (**c**) OPSF, and (**d**) CSOF approach; cut–off = 0.025 mm.

**Figure 14:**
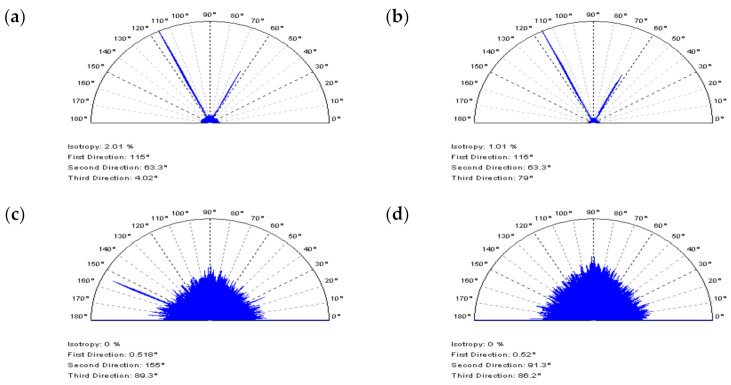
Texture direction graphs of NSs received from the milled surface texture with the application of (**a**) CSSF, (**b**) OPGF, (**c**) OPSF, and (**d**) CSOF.

The initial assumption was that the application of CSSF or OPGF would cause a filtration (removal) of treatment features within milled textures. It is most clearly detectable when intently studying the threshold ACF plots—NS contains two dominant directions. All of the milled surface analyses are illustrated in Figure 13 and Figure 14. The non-noise features in NS are present when implementing OPSF or CSOF, however, the ACFs retain the periodic characteristics when using the correct method. The application of texture direction graph does not define the periodicity nor the dominant direction. Consequently, the CSOF algorithm may be a practical solution for the reduction of high-frequency errors in the measurement process of milled topographies.

**Figure 15:**
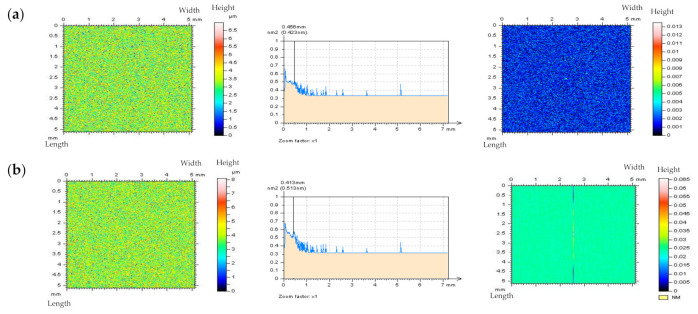

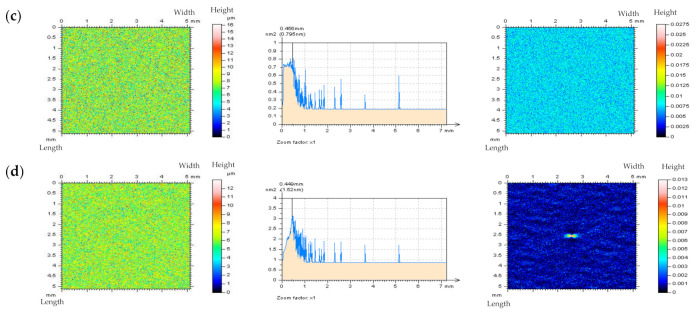
NSs, their PSDs, and threshold ACFs, respectively, identified for the composite surface topography measured with an optical technique, filtered by: (**a**) CSSF, (**b**) OPGF, (**c**) OPSF, and (**d**) CSOF procedure; cut–off = 0.025 mm.

**Figure 16:**
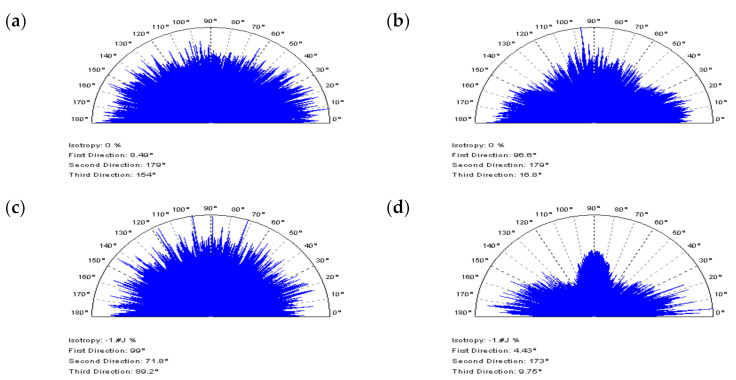
Texture direction graphs of NSs obtained for the composite surface texture with the application of (**a**) CSSF, (**b**) OPGF, (**c**) OPSF, and (**d**) CSOF.

When considering the non-periodic textures (e.g., composite, ceramic, isotropic in general), the non-HFN features are inaccessible in the optical detection process of NSs. Nonetheless, the disparities were apparent for PSD assays of compared spline filters. Thus, a multithreaded analysis of all of the proposed graphs is invaluable in the noise-separation selection procedure. The CSOF approach extracts the 0.45 mm frequency as dominant (in NS). Accordingly, the separated noise was not as high in frequency as required. The same remark applies to the ACF assessments, in which the texture direction is also anisotropic (Figure 15 and Figure 16). The OPGF method has one dominant direction for both: ACF and texture direction plots. The NS achieved by the filtering of isotropic textures should be non-periodic in general.

The differences between the application of CSSF and OPSF algorithms lay mostly in the noise amplitude. However, the HFN amplitude is not the focus of this paper. Both filters are suitable for the reduction of high-frequency errors from the measurement results of non-periodic textures, e.g., ceramic or composite surface topographies.

Generally, the denoising (of the HFN) from the measurement results of various types of textures should be performed with multithreaded aspects, taking into consideration various aspects, as mentioned above.

### 3.3. Improving Procedures with a Modelled Data

In previous studies, the influence of the occurrence of HFN on the surface topography parameters was analyzed. It was directly observed that the analyzed type of measurement errors largely affect some of the parameters. The values of the maximum height of surface *Sz*, root-mean-square gradient (slope) *Sdq*, peak density *Spd,* and core roughness depth (core height) *Sk* usually increased more than 100% when the *Sq* of noise was greater than 20% of *Sq* of the analyzed surface. In certain instances, the observed differences reached even 1000% [100]. The four parameters mentioned above were described as Noise Parameters (NPs)—the most sensitive parameters of HFN occurrence.

Furthermore, it is valuable to observe the variations of values of surface topography parameters when the reduction of high-frequency errors are considered. One should compare the absolute differences of NPs values, calculated for measured detail, and subsequently, the same detail after adding a modelled noise and its removal. Moreover, analyses of the NPs (and other surface texture parameters) for received NS can also be proposed.

The calculation of the *NPs* relative differences sum, and the other parameters’ relative differences sum (non-*NPs*, short *nNPs*—all other parameters, excluding *NPs*) is suggested. The value of the first sum should be maximized, while simultaneously, the second should be minimized. The proposed procedure can be described as follows:

(21) $$\left\{\begin{array}{c} \left|\Delta NP\right|\to \underset{NP}{\max}\\ \left|\Delta nNP\right|\to \underset{nNP}{\min} \end{array}\right.$$

where

(22) $$\left|\Delta NP\right|=\sum \left|\Delta NPs\right|$$

and

(23) $$\left|\Delta nNP\right|=\sum \left|\Delta nNPs\right|.$$

The following equation may equal the *NPs* sum value

(24) $$\sum \left|\Delta NPs\right|=\left|\Delta Sz\right|+\left|\Delta Sdq\right|+\left|\Delta Spd\right|+\left|\Delta Sk\right|$$

and the sum of the rest of the parameters

(25) $$\sum \left|\Delta nNPs\right|=\left|\Delta HP\right|+\left|\Delta FP\right|+\left|\Delta SP\right|+\left|\Delta HYP\right|+\left|\Delta FEP\right|+\left|\Delta FUP\right|$$

where

(26) $$\left|\Delta HP\right|=\left|\Delta Sq\right|+\left|\Delta Ssk\right|+\left|\Delta Sku\right|+\left|\Delta Sp\right|+\left|\Delta Sv\right|+\left|\Delta Sa\right|$$

(27) $$\left|\Delta FP\right|=\left|\Delta Smr\right|+\left|\Delta Smc\right|+\left|\Delta Sxp\right|$$

(28) $$\left|\Delta SP\right|=\left|\Delta Sal\right|+\left|\Delta Str\right|+\left|\Delta Std\right|$$

(29) $$\left|\Delta HYP\right|=\left|\Delta Sdr\right|$$

(30) $$\left|\Delta FEP\right|=\left|\Delta Spc\right|$$

(31) $$\left|\Delta FUP\right|=\left|\Delta Spk\right|+\left|\Delta Svk\right|.$$

**Figure 17:**
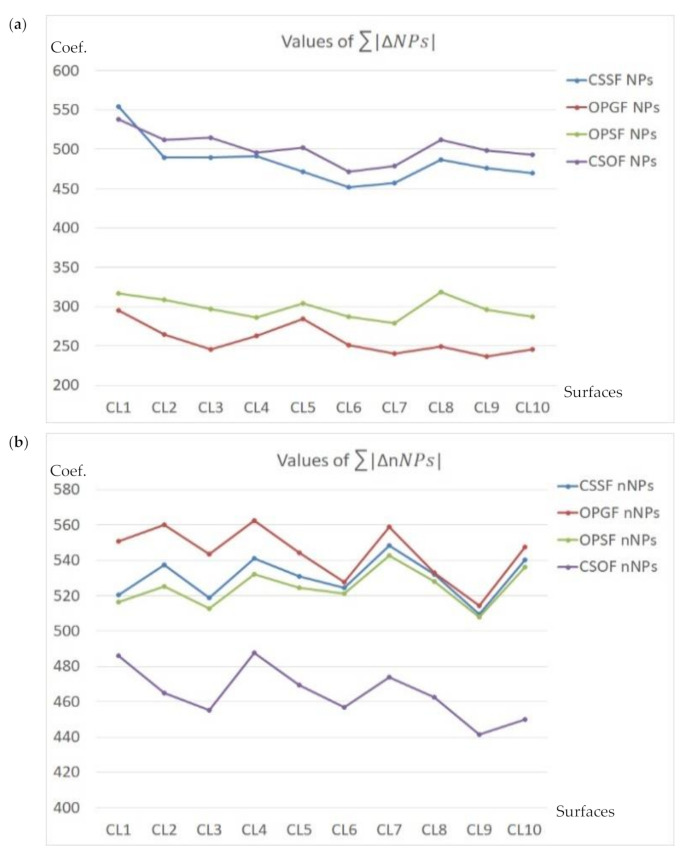
The values (presented in Coef. axis) of sums of NPs (**a**) and nNPs (**b**), described for plateau-honed cylinder liner surfaces.

**Figure 18:**
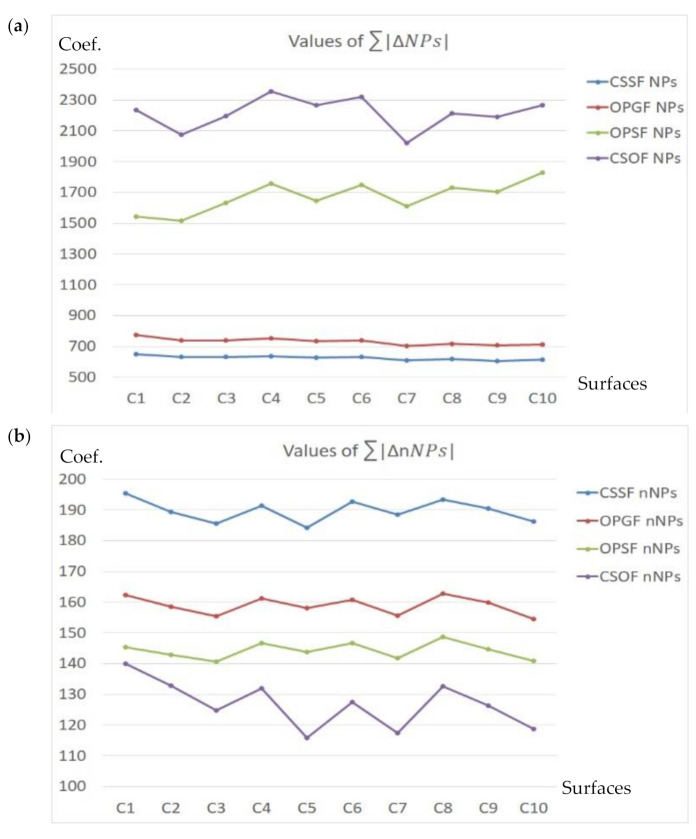
The values (presented in Coef. axis) of sums of NPs (**a**) and nNPs (**b**), described for plateau-honed cylinder liner surfaces.

**Figure 19:**
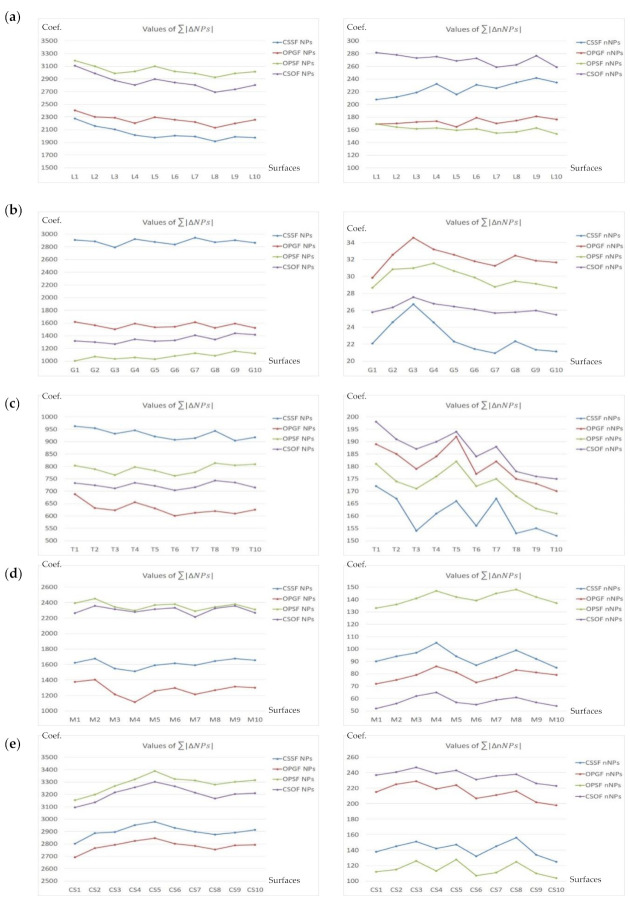
The values (presented in Coef. axis) of sums of NPs and nNPs, respectively, described for laser-textured (**a**), ground (**b**), turned (**c**), milled (**d**), and composite (**e**) surfaces.

The *NPs* value should be altered the most. In most cases, primarily the peak density (*Spd*) increased. Ten surfaces were analyzed, ten for each type of texture, as follows: Plateau-honed cylinder liners (marked CL1–CL10), ceramic (C1–C10), laser-textured (L1–L10), ground (G1–G10), turned (T1–T10), milled (M1–M10), or composite (CS1–CS10) surfaces. In Figure 17, Figure 18 and Figure 19, the values of NPs and nNPs were presented, respectively. The *X*-axis indicated each of the analyzed surfaces, *Y*-axis the values of the *NPs* and *nNPs* factors, respectively. In Figure 17, the values of both sums are presented for plateau-honed cylinder liners surfaces. If relations (21) are resolved for the honed cylinder textures, the CSOF gives the most favourable response for the extraction and reduction of the HFN from the measured results. The application of CSSF gave similar results for the considered relation of the NPs sum, but the minimization of the nNPs sum was far from satisfying. CSOF can also be directly applied for noise reduction in the process of ceramic surfaces measurement (Figure 18).

In Figure 19, the sum of the relative differences in *NPs* and *nNPs* were presented. OPSF gave the most satisfying results amongst the laser-textured (a) surfaces, both relations from Equation (21) were successfully met. When considering the ground (b) or turned (c) details, the application of the CSSF method provided the expected response. The milled (d) texture maximization of the *NPs* sum was obtained for OPSF. However, the minimization of nNPs was not achieved and the value of *nNPs* was maximized. Therefore, another filter should be considered. The in-depth analysis proved that the CSOF approach gives comparable results for the NPs sum. As it was minimized, the CSOF procedure consequently became more relevant than the OPSF from the process of reducing the HFN from the milled surface texture measurements. The best solution for composite (e) surface topographies from the proposed and compared spline filters is clear. The NPs maximization and *nNPs* minimization were achieved when using OPSF. Consequently, this type of surface topography S-operator filtering method is the suggested approach for the reduction of HFN from the raw measured data.

The proposed procedure may be applied to various types of surface textures or filtration methods. It is not only the spline function/filter that can be thoroughly evaluated in terms of the practicality of various algorithms, intended for the reduction or minimization of HFN influence.

## 4. The Outlook

Despite having compared the various types of noise detection procedures (free of-dimple analysis, profile spline method, autocorrelation function centre-shape method, treatment trace-profile method, or other approaches of noise detection, e.g., the procedure for laser-textured surface topographies) and spline filters, both intended for the reduction of high-frequency measurement errors, there are still many aspects that deserve thorough analysis. The particularly resinous concerns can be specified, as follows:

1. Primarily, the influence of wear on the occurrence of high-frequency measurement noise (and vice versa), which was not considered in this paper and deserves close attention in further research, as does the HFN for worn surface (details).
2. The presented procedures for noise estimation might not be sufficient, outside of simulation tests. Furthermore, the recommended approaches might prove to be futile in cases when the amplitude errors are too small to eradicate. Moreover, some non-noise (in particular, non-HFN) components are also directly exposed to raw measured data when S-filtration is accomplished.
3. There should also be a proposed research method for cases in which the raw data received is compared with the results which were obtained while the measurement is relatively small, e.g., 0.5 mm/s. Subsequently, the difference between fast measurement (e.g., 1.5 mm/s) after filtration (i.e., using spline schemes) and raw measured data with a low velocity (e.g., 0.5 mm/s), should be minimized. The smaller the difference separating the results of the processed fast measurement (for HFN reduction) and the unprocessed results measured at a slower pace, the better. There is a considerable advantage of the procedure.
4. The NPs were defined as common “noise parameters” for all of the studied details. Nonetheless, the number of NPs can be slightly or, in some cases, considerably enlarged for each type of the analyzed textures. Thus, the non-general designated NPs and factors might be more commonly useful, considering their relative difference changes.
5. A serious problem remains with edge-effect minimization in surface topography measurements. This type of data processing errors method is especially concerned with the extraction of waviness from the raw data. The edge limitations increase when deep or wide features (e.g., dimples, scratches, oil pockets, or valleys in general) are situated near the edge of a studied surface topography detail. The influence of density and amplitude of errors on the sides (edges) of the measured component on the HFN occurrence and their effect on the surface texture parameter calculation were not fully analyzed. Sharp-edge noise can also be considered for sharp-edged features, such as deep holes. However, the results of the received (extracted) noise (HFN in general) might be of different amplitudes in each NS area. The noise gathering process was also omitted.
6. Establishing the locations in which the noise is easy to detect constitutes another challenge. Free-of-dimple or profile spline methods are typical examples of this form of HFN detection. Moreover, the noise amplitude might be falsely estimated when non-feature details are considered, as opposed to the in-depth valley analysis. Distinguishing the studies involving valley-part or plateau-part might prove useful for noise detection but not for the minimization of the considered frequency errors. Therefore, much attention should be directed to the analysis of the entirety of the measured detail.

## 5. Conclusions

Definition and minimization of the high-frequency measurement errors are complicated, nevertheless, from the presented studies the following remarks can be provided:

1. It was found that when the number of features (e.g., dimples, valleys, scratches) increased in the plateau-honed cylinder liner surfaces, the accuracy of the high-frequency noise detection by optical analysis of Power Spectral Density graphs decreased. Consequently, the chosen solution was an out-of-feature analysis of the specific details. The optical detection of noise was more effective during the 2D (profile) analysis than in the 3D (areal) surface topography assessment. The paper proves that in the plateau-honed cylinder liner surface containing additionally burnished dimples, the detection of high-frequency noise is entirely dependent on the valley occurrence. When a certain profile contains only scratches (of a width < 50 um) and their number (density) is relatively small (profile < 3 mm, length equal to 4 or 5 mm), the detection of noise using the Power Spectral Density plot analysis becomes possible.
2. When considering the valley profiles (profiles with dimples), the problems with noise detection using the PSD graph analysis increased. Severe problems with noise detection appeared in cases with the largest number (density) of dimples. Therefore, the out-of-dimple (valley or scratch) detail (and, usually, profile) analysis is required for the detection of high-frequency noise within the raw measured data, rather than the entire measured detail assessment.
3. An alternative is the profile spline method. It is proposed rather than the out-of-feature (e.g., dimple) noise detection. This approach extracts the out-of-valley parts of the profile from the raw measurement profile data and connects them. The free-of-dimple profile is not required for this type of examination. The plateau-and-valley extraction accuracy was not studied in detail in this paper. However, some papers already cover the efficiency of various distinguishing methods.
4. Similar to the out-of-dimple method is a “treatment trace-profile” analysis, where the studied profiles are extracted along the trace of treatment received directly from the manufacturing process. This type of analysis can be applied to other kinds of surface textures than the plateau-honed topographies. The “treatment trace profile” analysis is particularly valuable, in some cases, for turned or ground details assessments.
5. According to the applied approach, each spline filtering method is adequate for the noise extraction of various types of surface textures, e.g., Open Profile Spline Filter seems to be the most useful in the analysis of laser-textured or composite surfaces, Cubic Spline Outlier Filter is valuable in studying the honed, ceramic, or milled topographies, Cubic Smoothing Spline Filter gave encouraging results for the turned or ground details. The suggested minimization approach can also be applied for other types of surface topographies. Additionally, alternative types of filtering methods may be used for the characterization of engineering surfaces. The achieved results can also be compared with those presented in recent studies.
6. Finally, high-frequency noise analysis with multithreaded intention is suggested. The analysis of the Power Spectral Density and the Autocorrelation Function graphs can provide much valuable information about noise identification, especially with regard to the analysis of “noise surface” (defined as a result of the noise-removal S-filtering process). The proposal was that “noise surface” should only contain the frequencies needing to be removed, i.e., after the high-frequency noise reduction process, the received noise data (NS) should only consist of high-frequency components, non-noise features should not be present. When other unrequired surface features are present, the less popular approach (filter) is suitable for the reduction of high-frequency measurement errors.

In the future, the reviews of recent filtration techniques for both wavelets and spline methods will be published by the author.

## Data Availability

Data sharing is not applicable to this article.

## Nomenclature

The following abbreviations with formulas designations (left column) and parameters (right column) are used in the manuscript:

*ACF*	autocorrelation function	*Sa*	arithmetic mean height Sa, µm
*ACF-CSM*	Autocorrelation Function Centre-Shape Method	*Sal*	auto-correlation length, mm
*CPSF*	Close Profile Spline Filter	*Sbi*	surface bearing index
*CSSF*	Cubic Smoothing Spline Filter	*Sci*	core fluid retention index
*EDM*	Empirical Mode Decomposition	*Sdq*	Root-mean-square gradient
*FOD*	free of dimple (valley) surface or profile Gauss	*Sdr*	developed interfacial areal ratio, %
*FFTF*	Fast Fourier Transform Filter	*Sk*	core roughness depth, µm
*HFN*	high-frequency noise	*Sku*	kurtosis
*MA*	multithreaded approach	*Sp*	maximum peak height, µm
*MQCL*	Minimum Quantity Cooling Lubrication	*Spc*	arithmetic mean peak curvature, 1/mm
*NURBS*	non-uniform rational B-spline surfaces	*Spd*	peak density, 1/mm^2^
*OPGF*	Open Profile Gaussian Filter	*Spk*	reduced summit height, µm
*OPSF*	Open Profile Spline Filter	*Sq*	Root-mean-square height, µm
*PSD*	power spectral density	*Ssk*	skewness
*PSM*	Profile Spline Method	*Std*	texture direction, °
*PSWF*	Polynomial Spline Wavelet Filter	*Str*	texture parameter
*RSM*	root-mean-square	*Sv*	maximum valley depth, µm
*TTP*	treatment-trace-profiles approach	*Svi*	valley fluid retention index
*WLIAFM*	white light interference-based atomic force microscopy	*Svk*	reduced valley depth, µm
*ω*	vector of output data values	*Sz*	the maximum height of surface, µm
$\lambda_{c}$	cut-off value	*Ra*	arithmetic mean deviation of rough. profile, µm
*s(x)*	the weighting function	*Rc*	mean height of roughness profile element, µm
f	a “noisy-data” vector	*Rdc*	roughness profile Section height difference, µm
$f_{i}$	the example of “noisy data”	*Rdq*	root-mean-square slope of roughness profile, °
$g\left(x\right)$	cubic spline smoothing curve	*Rmr*	relative material ratio of roughness profile, %
g	a smoothed vector	*Rp*	maximum peak height of roughness profile, µm
A	a symmetric three-diagonal matrix with the non-null elements of the ith row or column	*RPc*	peak count of the raw profile, 1/mm
$c\left(k\right)$	the B-spline coefficients	*Rq*	root-mean-square (RSM) dev. of r. profile, µm
$B^{0}\left(x\right)$	the indicator function	*RSm*	mean width of roughness profile element, mm
$B^{n}\left(t\right)$	the B-spline function constructed by a repeated convolution of a B-spline of order 0	*Rt*	total height of roughness profile, µm
$\beta^{n}\left(t\right)$	the central B-spline	*Rv*	maximum valley depth of roughness profile, µm
$\psi_{m}\left(t\right)$	the B-spline waelet	*Rz*	maximum height of roughness profile, µm
$N_{m}$	the mth order B-spline		
$s\left(x,y\right)$	the bi-cubic spline element		
c	the coefficients $c_{ij}$		
z	the values of $z_{r}$		
A	the Kronecker product of two-band matrices of small size		
